# Efficacy of high‐level disinfection of endoscopes contaminated with *Streptococcus equi* subspecies *equi* with 2 different disinfectants

**DOI:** 10.1111/jvim.16740

**Published:** 2023-05-26

**Authors:** Veridiana Nadruz, Laurie A. Beard, Katherine M. Delph‐Miller, Robert L. Larson, Jianfa Bai, Muckatira M. Chengappa

**Affiliations:** ^1^ Department of Clinical Sciences Kansas State University Manhattan Kansas USA; ^2^ Department of Diagnostic Medicine/Pathology Kansas State University Manhattan Kansas USA

**Keywords:** accelerated hydrogen peroxide, infection control, ortho‐phthaldehyde, polymerase chain reaction

## Abstract

**Background:**

Prevention of spread of *Streptococcus equi* subspecies *equi* (*S. equi*) after an outbreak is best accomplished by endoscopic lavage of the guttural pouch, with samples tested by culture and real time, quantitative polymerase chain reaction (qPCR). Disinfection of endoscopes must eliminate bacteria and DNA to avoid false diagnosis of carrier horses of *S. equi*.

**Hypothesis/Objectives:**

Compare failure rates of disinfection of endoscopes contaminated with *S. equi* using 2 disinfectants (accelerated hydrogen peroxide [AHP] or ortho‐phthalaldehyde [OPA]). The null hypothesis was that there would be no difference between the AHP and OPA products (based on culture and qPCR results) after disinfection.

**Methods:**

Endoscopes contaminated with *S. equi* were disinfected using AHP, OPA or water (control). Samples were collected before and after disinfection and submitted for detection of *S. equi* by culture and qPCR. Using a multivariable logistic regression model‐adjusted probability, with endoscope and day as controlled variables, the probability of an endoscope being qPCR‐positive was determined.

**Results:**

After disinfection, all endoscopes were culture‐negative (0%). However, the raw unadjusted qPCR data were positive for 33% AHP, 73% OPA, and 71% control samples. The model‐adjusted probability of being qPCR‐positive after AHP disinfection was lower (0.31; 95% confidence interval [CI], −0.03‐0.64) compared to OPA (0.81; 95% CI, 0.55‐1.06), and control (0.72; 95% CI, 0.41‐1.04).

**Conclusion and Clinical Importance:**

Disinfection using the AHP product resulted in significantly lower probability of endoscopes being qPCR‐positive compared to the OPA product and control.

**Abbreviations**
AHP: accelerated hydrogen peroxide
BHI: brain heart infusion
CT: cycle threshold
OPA: ortho‐phthaldehyde
*S. equi*
: *Streptococcus equi* subspecies *equi*
VDL: Veterinary Diagnostic Laboratory

## 1 INTRODUCTION

*Streptococcus equi* subspecies *equi* (*S. equi*) causes strangles, a worldwide infectious disease in horses that results in substantial morbidity. Carrier horses, which can be clinically normal, harbor the organism in their guttural pouch, resulting in additional outbreaks.^1^, ^2^ Detection of carrier horses is best accomplished by endoscopic examination and guttural pouch lavage, with samples tested by culture or real time, quantitative polymerase chain reaction (qPCR).^1^, ^3^

Devices that come into contact with mucous membranes, such as endoscopes, are considered semi‐critical devices, which must undergo high‐level disinfection between uses, because contaminated endoscopes are linked to outbreaks of nosocomial infections in human medicine.^4^, ^5^, ^6^ Only 1 published study has evaluated the efficacy of disinfection of contaminated endoscopes in veterinary medicine.^7^ Most endoscopes were culture‐negative, after several different methods of disinfection after experimental contamination with *S. equi*.^7^ However, qPCR results were positive for 2/6 endoscopes cleaned with an enzymatic cleaner followed by disinfection using an ortho‐phthalaldehyde (OPA).^7^ Researchers concluded that horses could be falsely diagnosed as strangles carriers based on these results.^7^

It is common for veterinarians to perform multiple endoscopic examinations on several horses in a field setting after an outbreak, making manual high‐level disinfection necessary. Currently, the disinfectants most currently used are OPA products, which require 12 minutes of contact time with the endoscope.^8^ Commercial hydrogen peroxide is another broad‐spectrum antimicrobial, but most products containing hydrogen peroxide are highly corrosive to endoscopes. Accelerated hydrogen peroxide (AHP) is a newly developed product that is a blend of 2% hydrogen peroxide, surfactants, and stabilizers.^9^ This AHP product is compatible with flexible endoscopes, and only requires an 8‐minute contact time.^9^ Using a disinfectant with a shorter contact time would be beneficial to clinicians who need to perform several endoscopic examinations and disinfect the endoscope between several procedures in a single day.

The qPCR is currently considered the gold standard for detection of *S. equi*, even if culture is negative.^1^ A commonly asked question when horses are positive using qPCR test results alone is, “What is the possibility that this finding is real or could it be a result of contamination?” It appears from previous studies that iatrogenic spread of *S. equi* by disinfected endoscopes is unlikely, but disinfection does not always remove all DNA.^7^ Our objective was to compare failure rates (using qPCR and culture) of a high level of manual disinfection of endoscopes for *S. equi* using AHP and OPA products. The null hypothesis was that no difference would be found between the AHP and OPA products with regard to culture and qPCR results after disinfection.

## 2 MATERIALS AND METHODS

Two different disinfectant products were tested to determine the efficacy of manual high‐level disinfection of endoscopes using: (a) AHP (Prevention HLD Virox Technologies, Ontario, CA); (b) OPA (Metricide OPA Plus, Metrex, Orange, CA); and (c) control (water). Endoscopes were contaminated and disinfected 30 times with each disinfectant using a randomized block design (15 disinfections performed per day for 6 days). Two different endoscopes were used, which were leak tested at the start of each testing day. The endoscopes were disinfected twice using a new OPA product the day before each testing day and then tested by qPCR (as described below).

### 2.1 Contamination and sample collection

A broth culture of *S. equi* was prepared by adding 1 colony of wild type *S. equi* (obtained from Kansas State Veterinary Diagnostic Laboratory [VDL] Manhattan, KS) into 15 mL of brain heart infusion (BHI) broth and incubated at 37°C for 15‐18 hours. The same wild type *S. equi* strain was used throughout the study. Before starting the experiment, *S. equi* was confirmed by testing 1 colony on a plated and incubated sample, using matrix‐assisted laser desorption of ionization‐time of flight mass spectrometry for microbiological identification and qPCR. For each testing day, 5‐15 mL BHI *S. equi* broths were prepared. Bacterial growth was >100 000 colony forming units (cfu)/mL. The distal end of the endoscope was placed in a 50 mL conical tube. Five milliliters of *S. equi* broth was pipetted over the distal end of the endoscope and accumulated in the bottom of the conical tube. A sterile endoscopic catheter was passed through the biopsy channel until it emerged from the distal end of the endoscope. Saline (30 mL) was flushed through the catheter, and the fluid was collected into the conical tube while the distal 5 cm of the endoscope remained immersed in the contaminated saline in the conical tube. The saline was aspirated back into the syringe, then flushed back through the catheter, and finally collected back into the conical tube (with the tip of the endoscope remaining in the saline‐filled conical tube for 5 minutes). The conical tube with the contaminated saline was submitted for culture and qPCR testing.

### 2.2 Disinfection

Three separate stations were set up, 1 for each disinfectant product. Each station had 4 different tubs containing (a) enzymatic cleaner (Endozime AW Triple Plus with APA, Ruhof, Mineola, NY), (b) water, (c) disinfectant, and (d) rinse water. The endoscopes first were immersed in the enzymatic cleaner (10 L), then water (10 L), then disinfectant (3.8 L), and finally in rinse water (10 L). The exterior of the endoscope was manually cleaned using a 4 × 4 gauze sponge in each tub. One metal‐non‐disposable brush was used for each disinfection station. The interior channels of the endoscopes were cleaned twice using endoscopic brushes and irrigated twice with either enzymatic cleaner (enzymatic tub), water (water tub) or disinfectant (disinfectant tub) using a 60‐mL syringe in each tub. A new 60‐mL syringe was used each time the endoscopes were disinfected. The endoscopes and the interior channels were completely immersed into each disinfectant for 12 minutes for the OPA^8^ and control and 8 minutes for the AHP.^9^ Manufacturer‐provided test strips were used to test the OPA and AHP to ensure adequate disinfectant concentration each time the endoscope was disinfected.^8^, ^10^ After the disinfectant, the endoscopes then were immersed into the final tub (rinse water) and all lumens were irrigated 3 times for a total of 180 mL per channel. After being immersed into water and irrigated, the endoscopes were rinsed (exterior and interior) using 70% isopropyl alcohol. The endoscopes were dried using a 4 × 4 gauze sponge and air was injected through the interior channels. Sample collection after endoscope disinfection was identical to sample collection after contamination (ie, using a conical tube, endoscopic catheter, and 30 mL of saline). Enzymatic cleaning solution and water in each tub were discarded and replaced each time between disinfecting each endoscope. All of the water used was municipal water. The disinfectants (AHP and OPA) were used for only 1 testing day and replaced with a fresh solution at the start of each new testing day.

It was not possible to blind personnel performing the contamination, disinfection, and sample collection because of the differences in time and smell for each disinfectant. In an attempt to prevent contamination of disinfected endoscopes, a single person contaminated endoscopes and collected samples after contamination for all 6 testing days. The same 2 people disinfected the endoscopes, and only 1 person collected the sample after disinfection for all 6 testing days. All personnel wore disposable protective gowns, gloves (double‐gloved), and plastic boots. The personnel disinfecting the endoscopes and collecting samples after disinfection changed protective clothing every time an endoscope was disinfected. At the end of each testing day, all equipment (tubs, tables, floor, and walls) were rinsed with water, disinfected using a different AHP product (Intervention Farm Animal Care, Virox Technologies, Ontario, CA) product used by the hospital, and allowed to air dry for at least 7 days.

Endoscope testing included aerobic culture and qPCR for *S. equi*, performed after contamination and after disinfection. The conical tubes were centrifuged at 3320 g for 15 minutes. After centrifugation, approximately 25 mL of supernatant was removed. Culture was performed by plating 1 μL of the pellet directly onto blood agar plates (tryptic soy agar with 5% defibrinated sheep blood), and incubated in a 5% CO_2_ atmosphere at 37°C for 24 hours. The remaining saline fluid was frozen at −70°C until qPCR could be performed. The *SeM* gene that codes for the antiphagocytic M protein of *S. equi* was used in the qPCR assay. The primers and the probe sequences were previously published.^11^ The assay specifically detects the pathogen and does not cross‐react with closely related pathogens including *S. equi* subsp. *zooepidemicus*. The assay has been fully validated analytically, and diagnostically validated using 42 positive and 280 negative clinical samples, with sequencing confirmation of the 25 positive samples published.^11^ The remaining 17 samples were sequenced later (unpublished data). The cycle threshold (CT) cutoff was set to 39 (>40 being negative). The assay currently is an official diagnostic test at the VDL. Personnel performing culture and qPCR were blinded to which disinfectant was used.

### 2.3 Statistical analysis

To provide evidence treatment differences for rare binomial events (ie, failure to sterilize; probability [P] of failure = 0.01), sample size was computed using the equation: *N* = (*Z*_*α*_ + *Z*_*ß*_)^2^ (*P*_*S*_ [1 − *P*_*S*_] + *P*_*T*_ [1 − *P*_*T*_]), where N = sample size for each treatment group, *Z*_*α*_ = standard normal variate corresponding to the *α* significance level (0.05), *Z*_*ß*_ = standard normal variate corresponding to the tail probability of size *ß* (0.10), *P*_*S*_ = probability of failure to sterilize using the standard treatment (OPA), *P*_*T*_ = probability of failure to sterilize using the comparison treatment, and the difference between the standard and new treatment effects that was considered clinically meaningful for the study was .075. The sample size calculated when using these assumptions was 30 replications per treatment. Using multivariable logistic regression model‐adjusted probability, with endoscope and day as control variables, the risk of an endoscope being qPCR‐positive after disinfection was determined.

## 3 RESULTS

**FIGURE 1:**
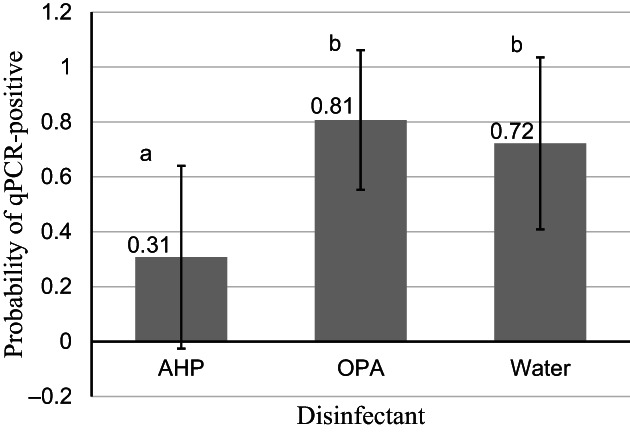
Probability (±95% CI) of endoscopes contaminated with *S. equi* being quantitative polymerase chain reaction (qPCR)‐positive disinfection with accelerated hydrogen peroxide (AHP), ortho‐phthaldehyde (OPA) or water (control). Letters a and b signify significant differences (*P* = .003). Disinfection with AHP resulted in a significantly lower probability of being qPCR‐positive after disinfection compared to OPA or water.

**FIGURE 2:**
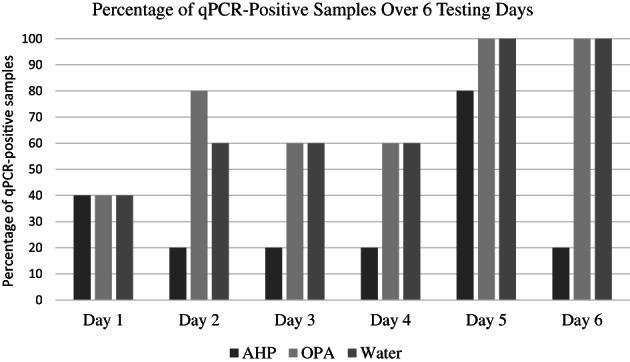
Percentage of quantitative polymerase chain reaction (qPC)‐positive samples following disinfection with accelerated hydrogen peroxide (AHP), ortho‐phthaldehyde (OPA), and Water over 6 Testing Days.

**FIGURE 3:**
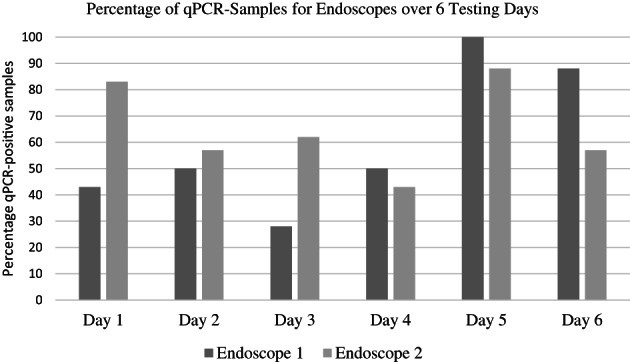
Percentage of quantitative polymerase chain reaction (qPCR)‐positive for endoscopes 1 and 2 over 6 testing days.

All samples were culture and qPCR‐positive after contamination. All samples were culture‐negative after disinfection (0%). However, unadjusted qPCR data were positive for 10/30 (33%) AHP, 22/30 (73%) OPA, and 21/30 (71%) control samples. The disinfectants were different in regard to qPCR‐positive test results after disinfection. The model‐adjusted probability of being qPCR‐positive following AHP disinfection was lower (0.31; 95% confidence interval [CI], −0.03‐0.64) compared to OPA (0.81; 95% CI, 0.55‐1.06) and control (0.72; 95% CI, 0.41‐1.04; Figure 1). Significant variability was found in the number of qPCR‐positive samples for each testing day, with day 5 having 14/15 endoscopes testing positive (Figure 2). The mean ± SD CT values for the qPCR after contamination were 20.8 ± 2.5 for AHP, 22.3 ± 1.8 for OPA, and 21.8 ± 2.2 for control samples. The mean CT values for the qPCR after disinfection were 37.0 ± 0.8 for AHP, 35.8 ± 1.5 for OPA, and 35.6 ± 1.3 for control samples. Despite being disinfected twice using a new OPA solution before the start of each testing day, endoscope number 1 was positive at the start of Days 1, 3, and 5. Testing the endoscopes before Day 4 was omitted by mistake. Although endoscope number 1 was positive at the start of 3 testing days, endoscope number 2 was positive more often than endoscope number 1 at the end of the study (30/45 vs 23/45 times tested; Figure 3).

## 4 DISCUSSION

We documented that high‐level manual disinfection using either AHP, OPA or even water was successful at eliminating live *S. equi* bacteria (as detected by culture) from endoscopes, which is similar to what has been reported previously.^7^ However, none of the disinfectants used completely eliminated DNA after disinfection, again similar to a previous report.^7^ Surprisingly, disinfection using the AHP product resulted in significantly lower probability of the endoscope being qPCR‐positive compared to the OPA product and water. However, even with use of the AHP product, the disinfected endoscopes were still qPCR‐positive 33% percent of the time.

Significant variability of the qPCR tests was observed day to day, with Day 5 having the highest number of positive tests (>90%). The most likely reason for the high failure rate on Days 5 and 6 could be environmental contamination. The same isolation stall, tubs, tables and non‐disposable metal endoscopic brushes were used for all 6 testing days. The equipment was disinfected (and allowed to dry for 7 days) between testing days. Environmental sampling, inclusion of a negative control group, or both could have addressed this problem. Human error also could have played a role in regard to Day 5. During the project in the spring of 2020, the COVID pandemic resulted in the shutdown of all research, resulting in a delay between Days 4 and 5 (45 days). Testing personnel possibly were less efficient on Day 5 and could have made more mistakes resulting in contamination. Finally, the endoscopes used in the project were old and it is possible that damage to the biopsy channel could have increased bacterial attachment and DNA residue.^12^ Despite endoscopes being sampled before the start of each testing day (ie, before contamination), qPCR results were not available until the end of the study. Endoscope number 1 was positive 3 times before contamination (Days 1, 3 and 5), suggesting that this endoscope could have been problematic. However, endoscope number 2 was positive more often than endoscope number 1 at completion of the study.

Our study is similar to a previous report that detected residual DNA contamination of endoscopes after disinfection.^7^ However, our study had a much higher rate of qPCR‐positive samples (73% of the time) compared to the previous study (33%).^7^ As discussed above, contamination likely contributed to the high number of qPCR‐positive results. Use of a new disposable plastic endoscopic brush likely would decrease some of the contamination (when compared to non‐disposable brushes). False positives may be less likely in a field setting because only 1 of 14 disinfected endoscopes tested positive by qPCR from horses that were documented carriers.^7^ This finding could be a result of longer exposure time to the OPA because in the field setting endoscopes were in contact with the OPA for a longer period of time (20 minutes).^7^ The BHI broth used in our study had a high bacterial concentration (>100 000 cfu/mL), which could be higher than that found in carrier horses. In the field trial, only 5/14 horses were culture and qPCR‐positive, with the other 9 horses being only qPCR‐positive, but culture negative, which further suggests that some carrier horses may have very low bacterial numbers.^7^ Finally, more aggressive rinsing of the endoscope also could have decreased DNA contamination. The OPA manufacturer recommends that the endoscopes be rinsed 3 times after exposure to the disinfectant product, and the endoscopes were only rinsed once in our study.^8^

All culture results were negative after disinfection when AHP, OPA, and even water were used as disinfectants. The water used as a control was municipal water (not distilled), but did have 2‐4 mg/L of chloramine added as a disinfectant. The proposed mechanism of action of chloramine (with monochloramine being most common) is inhibition of protein‐mediated processes including bacterial transport of substrates, respiration, and substrate dehydrogenation. Monochloramine does not severely damage cell walls, or react strongly with nucleic acids.^13^, ^14^ The use of municipal water likely did contribute to samples being culture‐negative, but probably did not have a substantial effect on residual bacterial DNA. Based on our study and previous studies, it is extremely unlikely that transmission of *S. equi* occurs with endoscopes after high‐level disinfection.^7^ The CT values for the positive qPCR samples after disinfection were very high (all >35). Numbers of bacteria recovered on culture are proportional to the sensitivity of the qPCR, such that samples positive on qPCR with a CT > 34 are culture‐negative.^15^ According to the current American College of Veterinary Internal Medicine consensus statement, the recommendation is to consider horses that are qPCR‐positive (but culture‐negative) to be carriers of *S. equi* regardless of the CT values.^1^, ^15^ Therefore, even with the culture‐negative results and high CT values after disinfection, we cannot say with certainty that all live bacteria were removed.

The reason that the AHP disinfectant resulted in a significantly lower probability of having the endoscope test positive on qPCR compared to the OPA product is unknown. Hydrogen peroxide is an oxidizing agent and works by producing free hydroxyl radicals, a powerful oxidant, which can initiate oxidation and damage to nucleic acids, proteins and lipids.^16^ However, evidence suggests that hydrogen peroxide results in a ferryl radical formed from DNA‐associated iron, and not a hydroxyl radical.^17^ The OPA product is an aromatic aldehyde that results in amino acid interactions, cross‐linking and increased membrane permeability by cytoplasmic membrane damage.^18^ Interference at the DNA level was restricted to high OPA concentrations (>500 mg/L) when in contact with the OPA for 30 minutes.^18^ However, the concentration of the OPA used in our study was 0.6% (6000 mg/L) which should be capable of DNA damage but contact time was only 12 minutes. The longer duration of contact time of the OPA in the field study (20 minutes) may have resulted in more DNA damage, and decreasing qPCR‐positive test results.^7^ Perhaps if the OPA product was in contact with the endoscopes for a longer duration of time it might have performed similar to the AHP product.

Traditional hydrogen peroxide disinfectants have a higher concentration (7%) than used in the AHP product (2%). These traditional concentrated hydrogen peroxide products are corrosive to many medical instruments, including flexible endoscopes. The AHP product is reported to be much less corrosive to medical equipment such as endoscopes. The AHP product was tested with an endoscope for its compatibility by soaking the endoscope 1000 cycles for 5 minutes of contact time, with the endoscope being evaluated every 24 hours.^9^ No functional or material cosmetic damage was reported on the endoscope at any time.^9^ The particular product used (Prevention HLD) is no longer available. However, a similar product (Revital‐Ox Resert High Level Disinfectant, Steris, Mentor OH) is currently available and designed for use on flexible endoscopes.^19^ According to the manufacturer's data, material compatibility was tested through hundreds of cycles of exposure of this product to substrates commonly used in endoscopes (plastics and elastomers), with no reports of incompatibility.^19^ Despite this published information regarding compatibility of AHP products with endoscopes, not all endoscope manufacturers recognize these products as being compatible with their endoscopes.^20^ A customer letter from Olympus in 2014 stated that, “Olympus has not tested the compatibility of Revital‐Ox™ Resert High Level Disinfectant STERIS with Olympus endoscopy equipment. As a result, Olympus does not list this particular product as a compatible or incompatible product for reprocessing of Olympus endoscopy equipment.”^21^ Veterinarians are encouraged to check with their endoscopic manufacturer before the use of these products.

Finally, the OPA and AHP disinfectants have additional differences. The OPA products are considered much less toxic than the previously used glutaraldehyde products. However, exposure to OPA products has resulted in asthma in humans.^22^ Anaphylaxis also has been reported in patients after endoscopic examinations using endoscopes disinfected with OPA products.^20^, ^23^ The AHP product is considered much less toxic and with minimal eye or skin irritation reported in a rabbit model.^9^ However, the AHP product was more expensive than the OPA product at the time of our study. The AHP product cost approximately twice as much as the OPA product.

Our major finding and that of the previous study^7^ is the risk for false positive qPCR results caused by bacterial DNA contamination of endoscopes after disinfection, which could result in the misdiagnosis of a healthy horse as a carrier of *S. equi*. If horses are being tested for *S. equi* by qPCR, it is important to ensure that endoscopes are known to be qPCR‐negative before testing. Environmental contamination and errors made by personnel disinfecting endoscopes also could contribute to endoscope contamination after disinfection. Veterinarians should keep these issues in mind when performing multiple endoscopic examinations after a *S. equi* outbreak in a field setting, especially when it is not possible to test the endoscopes between horses. The use of AHP products in a field setting should be considered, because a significantly decreased probability of the endoscope testing positive after disinfection was found. However, veterinarians should contact their endoscope manufacturer before the use of this disinfectant, because warranties may not be covered if there is any evidence of corrosion of the endoscope when this product is used. On the other hand, if restricted to the OPA, longer contact time (20 minutes) may help decrease the number of false positive qPCR results.^7^ Finally, new updated qPCR assays are being used in human medicine and food safety that can distinguish between live and inactivated microbes (propidium monoazide combined qPCR tests).^24^, ^25^ Propidium monoazide is a photoreactive dye that has a high affinity for DNA, and only penetrates damaged cell membranes.^24^, ^25^ The ability to distinguish between live and dead organisms would be extremely useful in trying to determine if a horse (culture negative, but qPCR positive) is indeed a true carrier making residual DNA on endoscopes no longer a concern.

## CONFLICT OF INTEREST DECLARATION

Authors declare no conflict of interest.

## OFF‐LABEL ANTIMICROBIAL DECLARATION

Authors declare no off‐label use of antimicrobials.

## INSTITUTIONAL ANIMAL CARE AND USE COMMITTEE (IACUC) OR OTHER APPROVAL DECLARATION

Authors declare no IACUC or other approval was needed.

## HUMAN ETHICS APPROVAL DECLARATION

Authors declare human ethics approval was not needed for this study.
